# Persistent atrial fibrillation ablation: glimpsing the light ahead?

**DOI:** 10.1093/europace/euaf037

**Published:** 2025-02-17

**Authors:** Xiaofeng Hu, Luigi Di Biase, Xumin Hou, Xu Liu

**Affiliations:** Department of Cardiology, Shanghai Chest Hospital, Shanghai Jiao Tong University School of Medicine, No.241 West Huaihai Road, Xuhui District, Shanghai 200030, China; Department of Medicine, Division of Cardiology, Montefiore Medical Center/Albert Einstein College of Medicine, New York, USA; Department of Cardiology, Shanghai Chest Hospital, Shanghai Jiao Tong University School of Medicine, No.241 West Huaihai Road, Xuhui District, Shanghai 200030, China; Department of Cardiology, Shanghai Chest Hospital, Shanghai Jiao Tong University School of Medicine, No.241 West Huaihai Road, Xuhui District, Shanghai 200030, China

**Keywords:** Persistent atrial fibrillation, Ablation strategies, Electro-anatomical mapping, Pulse field ablation

## Abstract

Current ablation strategies for persistent atrial fibrillation (AF) remain suboptimal, with success rates around 50%. Pulmonary vein isolation serves as the cornerstone of ablation, yet adjunctive strategies have shown inconsistent results in randomized controlled trials. This review critically examines the outcomes and limitations of these approaches while identifying key barriers to success, including incomplete understanding of AF mechanisms, patient heterogeneity, technical challenges in achieving durable lesions, and the absence of standardized procedural endpoints. A novel electro-anatomically guided ablation protocol is proposed, integrating advanced mapping techniques and procedural endpoints aimed at achieving AF termination. Furthermore, it discusses emerging technologies such as pulsed field ablation, which hold promise for enhancing safety and long-term outcomes. These insights provide a framework for future research and the optimization of persistent AF ablation strategies.

## Graphical Abstract

**Graphical Abstract:**
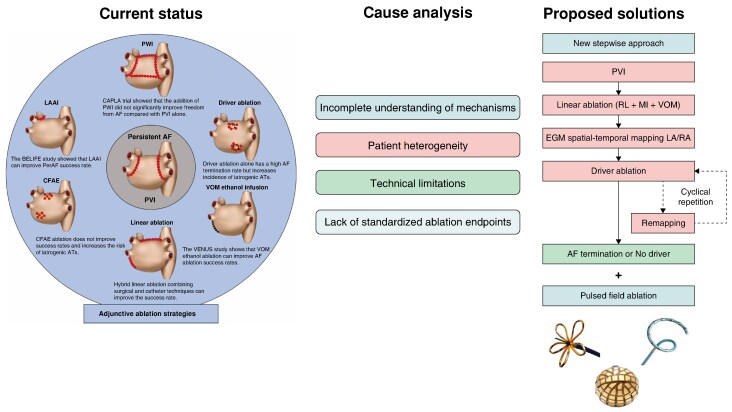

**What’s new?**

- Summarizes findings from randomized controlled trials on persistent atrial fibrillation ablation, highlighting key challenges such as mechanism uncertainty, patient heterogeneity, technical limitations, and lack of standardized endpoints.
- Analyses the efficacy and limitations of major ablation strategies, including linear ablation, complex fractionated atrial electrogram ablation, and driver ablation, identifying specific shortcomings.
- Proposes a new electro-anatomically guided ablation protocol, emphasizes the role of pulsed field ablation, and provides future directions for improving long-term outcomes.

## Introduction

Current therapeutic strategies for persistent atrial fibrillation (AF) yield limited success rates of ∼50%, underscoring the urgent necessity to enhance ablation outcomes.^1–5^ While pulmonary vein isolation (PVI) is considered the mainstay of AF ablation, two decades of advancement in adjunctive ablation strategies did not provide convincing evidence of better ablation outcome.^6,7^ A recent European Heart Rhythm Association survey highlighted the variability in clinical practice regarding ablation strategies for persistent AF across different centres, reflecting ongoing uncertainties and emphasizing the need for standardized protocols.^8^ In this paper, we summarized all randomized controlled trials (RCTs) on catheter ablation for the treatment of persistent AF over the past two decades. Surprisingly, only a small proportion of these studies have reported favourable outcomes. We conducted a thorough analysis to elucidate the reasons behind this observation. The poorly understood mechanisms sustaining AF contribute to the ineffectiveness of current ablation strategies in targeting these mechanisms, compounded by the absence of uniform ablative endpoints. This paper will delve further into this issue, incorporating insights on future directions to advance the field.

To evaluate the outcomes of different ablation strategies for persistent and longstanding persistent AF, a PubMed search was conducted using the terms ‘persistent atrial fibrillation’ OR ‘longstanding persistent atrial fibrillation’ OR ‘non-paroxysmal atrial fibrillation’ AND ‘ablation’. The search period was set from 1 January 2004 to 31 October 2024, and results were limited to clinical trials and randomized clinical trials. Studies were excluded if they were retrospective, irrelevant, or included patients with prior left atrial ablation. Additional relevant studies identified through manual searching were also considered for inclusion. The final selection of studies will provide a comprehensive assessment of the efficacy of various ablation strategies in patients with non-paroxysmal AF. Based on the aforementioned search criteria, 65 RCTs were included and served as the basis of this review (see Supplementary material online, *Table S1*).

## Mechanisms sustaining human atrial fibrillation

**Figure 1:**
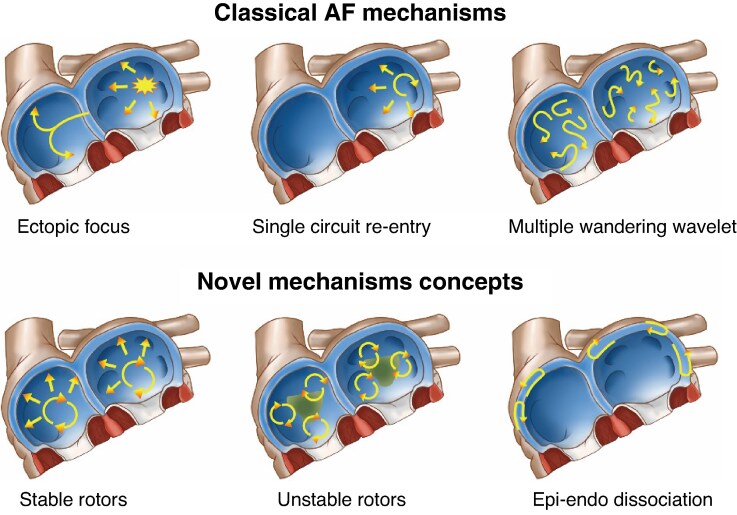
Mechanisms of AF. AF, atrial fibrillation.

In 1959, Moe *et al*.^9^ introduced a pioneering theory proposing AF as a stable, self-sustaining state independent of its initial triggers. Supported by experimental and computational data, their work underscored re-entry as the primary mechanism driving atrial fibrillatory activity.^10^ Allessie *et al*.'s^11^ studies in canine hearts further validated this hypothesis, demonstrating multiple propagating wavelets contributing to turbulent atrial electrical activity. Subsequently, the multi-wavelet mechanism was widely accepted.^12^ Clinical validation followed with the maze procedure by Cox and colleagues, which effectively prevented AF by creating isolated atrial regions incapable of sustaining re-entrant circuits.^13,14^ Recent research by Lee *et al*.,^15^ utilizing advanced mapping techniques, challenged the traditional multiple-wavelet theory, suggesting that AF may be driven by localized, repetitive focal sources spanning endo-epicardial layers. This ‘double-layer hypothesis’ posits continuous breakthroughs between atrial layers, potentially reconciling conflicting observations and highlighting the complexity of AF mechanisms (*Figure 1*).

Cardiac optical mapping with voltage-sensitive probes during the late 20th century significantly advanced our understanding of AF drivers. High-resolution optical mapping revealed that AF can be sustained by high-frequency rotors generating spiral (in 2D) or scroll (in 3D) waves interacting with cardiac tissue obstacles, leading to fragmentation and the formation of new wavelets.^16–18^ This theory suggests that AF maintenance involves sustained, functional re-entrant activity with complex wave propagation patterns, explaining the observed gradients of activation frequencies across atrial regions. Recent studies show that AF drivers can persist for months, often recurring at fixed locations and linked to fibrotic regions detected by late gadolinium enhancement magnetic resonance imaging, suggesting a structural basis for their stability.^19^ Despite debates over AF drivers and the variety of patterns in remodelled atria, the prevailing theory posits that AF is sustained by high-frequency drivers creating a hierarchy of progressively slowing activation rates as wavefronts spread outward from their origin.^20,21^ This concept has been translated from experimental studies to early clinical trials that target and ablate areas of highest activation to terminate AF.^22–24^

Haïssaguerre *et al*.^25,26^ first reported the pulmonary veins origin of paroxysmal AF, establishing a disease continuum from pulmonary veins triggers to paroxysmal AF and subsequently to persistent AF. Within this continuum, no study has yet elucidated how paroxysmal AF transitions into persistent AF. We propose that the generation of rotors is a key factor in this transition, with rotors driving rotors.^27,28^

## The evolution of ablation strategies

In 2005, Haïssaguerre *et al*.^29^ first described a stepwise ablation approach for persistent AF, which included PVI, complex fractionated atrial electrogram (CFAE) ablation, and linear ablation at the left atrial roof and mitral isthmus, aiming for AF termination as the endpoint. Initial reports demonstrated high success rates, with 87% AF termination and 95% sinus rhythm maintenance at 11 months. Despite the potential effectiveness of such procedures, the stepwise approach is characterized by prolonged procedural duration, excessive complexity, and a lack of precise ablation and hinders its widespread adoption.^30,31^ Furthermore, as procedural complexity increases, so do the risks of associated complications.^32^ However, subsequent RCTs (CHASE AF and Alster-Lost-AF Trial) have failed to demonstrate that the stepwise strategy achieves higher success rates compared with PVI alone.^33,34^

The STAR AF II trial aimed to compare different ablation approaches for persistent AF, initially assessing whether adjunctive techniques, such as the ablation of CFAEs or linear ablation, improve outcomes compared with PVI alone.^35^ Contrary to prevailing beliefs at the time, the STAR AF II trial revealed that additional ablation strategies did not improve freedom from AF or atrial arrhythmias compared with PVI alone. The overall success rate after one procedure was ∼50%. At that time, the understanding of CFAE and linear ablation techniques was insufficient.^36^ This limitation may explain why these additional ablation strategies did not result in improved outcomes. This point is corroborated by the latest EARNEST study, which, in the context of significantly reduced pulmonary vein reconnection rates, validated adjunctive left atrial linear ablation as superior to a standalone PVI strategy.^37^

Over the past decade, technologies have been developed to detect and target rotors. In 2012, Narayan *et al*.^38,39^ conducted a milestone study using a 64-electrode basket catheter with phase mapping to identify rotors in both left and right atria. Initial studies using this focal impulse and rotor modulation (FIRM) mapping technique reported promising results, demonstrating potential AF elimination in some patients. However, a subsequent multicentre study assessing the benefit of adding FIRM mapping to standard PVI found no significant improvement in AF outcomes compared with PVI alone.^40^ The subsequent randomized controlled OASIS study also showed no benefits from FIRM-guided ablation, noting an increase in complication rates and highlighting challenges in translating experimental data into routine clinical practice.^41^ Flaws identified with FIRM mapping included issues such as inter-spline bunching leading to a loss of coverage and contact, along with the procedural complexity.^42^ In 2017, Seitz *et al*.^43^ conducted a significant study using the PentaRay catheter to identify regions with rotor activity in AF, achieving high rates of AF termination and favourable clinical outcomes. Compared with Narayan's technique, this mapping method was more simplified and easier to implement in clinical practice. High-density mapping catheters like PentaRay offer advantages over FIRM mapping, providing higher resolution for pinpointing potential sources, as supported by computer simulation studies.^44^ However, the study methodology did not exclude fragmented potentials in the dispersion analysis, potentially targeting areas with both fragmented and non-fragmented potentials for ablation. Despite the high AF termination rate observed, concerns remain regarding the potential for unintentional pro-arrhythmic scar formation with extensive ablation.

In summary, there are four primary reasons limiting the success rate of catheter ablation for persistent AF: first, our understanding of the mechanisms responsible for AF initiation, maintenance, and progression remains incomplete; second, the heterogeneity of patients with persistent AF that were enrolled into RCTs thereby leading to inconsistent responses to ablation; third, technical limitations, such as the difficulty in achieving durable ablation lesions particularly linear lesion sets, compromise the long-term efficacy of ablation; and fourth, the absence of well-defined procedural endpoints for adjunctive ablation hampers the standardization of treatment success.

## Controversies in ablation strategies for persistent atrial fibrillation

Adjunctive ablation strategies for persistent AF beyond PVI can be categorized into three main types: (i) anatomical ablation: primarily linear ablation, including roof lines, mitral isthmus lines, and inferior lines; (ii) electrogram-guided ablation: targeting fragmented potentials, focal potentials, low-voltage substrate modification, and drivers; and (iii) electro-anatomical guided ablation: incorporating linear ablation alongside electrogram-guided ablation, such as stepwise approach.

Anatomical ablation, derived from the surgical maze procedure, aims to disrupt AF-sustaining re-entry circuits with electrical barriers, such as roof lines, mitral isthmus lines, and inferior lines alongside PVI.^45–47^ However, creating effective left atrial linear lesions remains technically challenging.^48^ Achieving bidirectional block is often difficult, increasing the risk of proarrhythmia from macro- or micro-re-entrant atrial tachycardias (ATs) utilizing gaps along the ablation lines.^49,50^ The STAR AF II study demonstrated that additional linear ablation, achieving a 74% rate of linear block, did not improve the success rate of persistent AF ablation.^35^ Although linear ablation is designed to mimic the surgical maze procedure, it falls short of achieving the same effect of atrial partitioning and the creation transmural lesions.^51^ This highlights the difficulties in replicating the durable barriers established surgically, which are critical for effectively disrupting the re-entry circuits that sustain AF.^52^ Consequently, linear ablation may not provide the same efficacy in long-term rhythm control and AF termination as the surgical maze procedure. Several randomized studies, including CONVERGE, CEASE-AF, and HARTCAP-AF,^53–55^ support this viewpoint by demonstrating that hybrid procedures incorporating surgical linear ablation techniques can significantly improve success rates in treating persistent AF (hybrid ablation vs. catheter ablation: 67.7–89% vs. 39.2–50%). The VENUS study indicated that additional vein of Marshall (VOM) ethanol infusion increased single ablation success rates, likely due to more durable peri-mitral block.^56^ The positive results can be attributed to the durable transmural blockades achieved by linear ablation. When excluding ATs from recurrent arrhythmic events, a meta-analysis indicated that linear lesions following PVI still do not significantly enhance AF-free survival in patients with persistent AF.^57^ Clinical studies have also shown that the termination rate of AF with linear ablation is relatively low, ∼20%.^58,59^ As one of the anatomical ablation strategies, posterior wall isolation has also yielded disappointing results in the CAPLA study, failing to demonstrate significant improvements in mid- or long-term outcomes.^60,61^ These observations suggest that anatomical ablation alone may achieve bidirectional block electrophysiologically but may not effectively modify the substrate that maintains AF. Thus, while linear ablation may modify atrial substrate, its ability to comprehensively address the multifactorial nature of AF remains uncertain and potentially inadequate for achieving long-term treatment success.

Due to the limitations of anatomical ablation, electrogram-guided ablation has emerged as an alternative treatment approach for persistent AF. Initial efforts targeted CFAEs, demonstrating potential for AF termination through focal triggers or micro-re-entrant circuits.^62^ However, the reproducibility of CFAEs ablation remains inconsistent.^3,63,64^ A rigorously defined visual scale for fractionation assessment has revealed that not all types of fractionated signals equally contribute to AF maintenance.^65,66^ Despite these insights, a consensus on the definition CFAE and which electrograms to ablate remains elusive.^67,68^ Extensive CFAE ablation can create non-contiguous lesion islands, potentially leading to slow conduction and widening of the excitable gap within the left atrium, thereby making adjunctive CFAE ablation a risk factor for post-procedural ATs.^69,70^ This suggests that CFAEs are passive areas of activation influenced by wavebreak dynamics, rather than active contributor to the maintenance of AF. Therefore, while ablating CFAEs may lack precision in targeting AF maintenance mechanisms, further mechanistic insights are crucial for determining the rationale behind continuing or discontinuing their therapeutic targeting. Furthermore, advancements in algorithm and mapping technologies continually enhance the accuracy of targeted electrogram (EGM) identification. These EGMs are identified as ‘drivers’ by characteristics such as spatial-temporal dispersion, high-frequency potentials, or locally short cycle lengths, indicating critical AF-maintaining regions.^71–73^ Driver ablation markedly improves the rate of AF termination compared with CFAE ablation,^58,74^ indicating its superior effectiveness in targeting the fundamental mechanisms of AF maintenance. Nevertheless, methodological heterogeneity has led to conflicting results despite these encouraging outcomes.^75,76^ The challenge of driver ablation lies in precisely identifying the critical drivers sustaining AF, which remains a pivotal issue in this field.^77^ Accurately identifying these drivers remains a significant hurdle. EGM-guided ablation, while precise, faces obstacles such as the absence of standardized EGM definitions and mapping methods, a steep learning curve, and the recurrence of AT.^78,79^ Clinical rotor identification is influenced by diverse surface activation patterns and inadequate mapping resolution, leading to operator-dependent outcomes.^73^ Many ablative procedures targeting drivers have not been consistently reproducible, potentially due to inherent drawbacks. This approach has been observed to create unnecessary proarrhythmic scars, significantly increasing the likelihood of recurrent AT. This highlights the difficulty of achieving effective ablation while mitigating adverse effects such as proarrhythmia.

Therefore, combining these two ablation strategies appears to be a judicious approach, leveraging their respective strengths to achieve more comprehensive outcomes. The classic stepwise approach, an electro-anatomically guided ablation strategy, shows a high arrhythmia-free survival rate post-multiple ablations and an impressive AF termination rate, underscoring its efficacy in extensive ablation.^29^ However, this method is associated with potential drawbacks, including the creation of unnecessary proarrhythmic scars and an increased risk of recurrent AT and complications.^80^ Furthermore, subsequent studies have struggled to consistently replicate these results.^33,34^ Therefore, an urgent need exists for a new electro-anatomical ablation strategy to address the aforementioned issues.

## Controversies in ablation strategies for persistent atrial fibrillation in heart failure patients

Atrial fibrillation combined with heart failure is a common clinical challenge. Multiple RCTs have demonstrated significant clinical benefits of catheter ablation in this population.^81–84^ However, the success rate of catheter ablation in remains relatively low, at ∼36%.^85^ The poor outcome is primarily due to the characteristics of these patients, including persistent AF, left atrial enlargement, significant atrial fibrosis, and marked electrical heterogeneity.^86,87^ Therefore, it is essential to identify the optimal ablation strategy for these patients.

In both the CASTLE-AF and CASTLE-HTx studies, a significant proportion of patients in the ablation groups had persistent AF, with some undergoing ablation beyond PVI.^82,84^ However, these studies lacked standardized ablation protocols, and the choice of additional strategies was left to the operator’s discretion, resulting in variability in approaches. Although promising results were observed, particularly with the PVI Plus strategy in the CASTLE-HTx study, the absence of direct comparisons between different ablation strategies highlights the ongoing lack of consensus on the optimal approach for this patient population.

Pulmonary vein isolation remains the cornerstone of AF ablation therapy, as recommended by current guidelines. However, evidence suggests that substrate-targeted ablation strategies can further improve outcomes in patients with persistent AF combined with heart failure. For instance, a stepwise approach achieving complete mitral isthmus block has been associated with ablation success rates as high as 72%.^88^ The AATAC trial demonstrated that PVI combined with posterior wall isolation resulted in better arrhythmia-free survival rates compared with PVI alone.^85^ Similarly, a subgroup analysis of the DECAAF II trial found that fibrosis-guided ablation in heart failure patients with persistent AF provided better arrhythmia-free survival outcomes than PVI alone.^89^ These findings emphasize the potential of substrate modification strategies to enhance outcomes in this patient population.

Therefore, additional ablation strategies based on PVI have shown potential to improve the success rates of catheter ablation in patients with heart failure and persistent AF. However, it is important to note that there is currently a lack of randomized studies specifically comparing different ablation strategies in this patient group.

## Controversies in clinical study design

**Figure 2:**
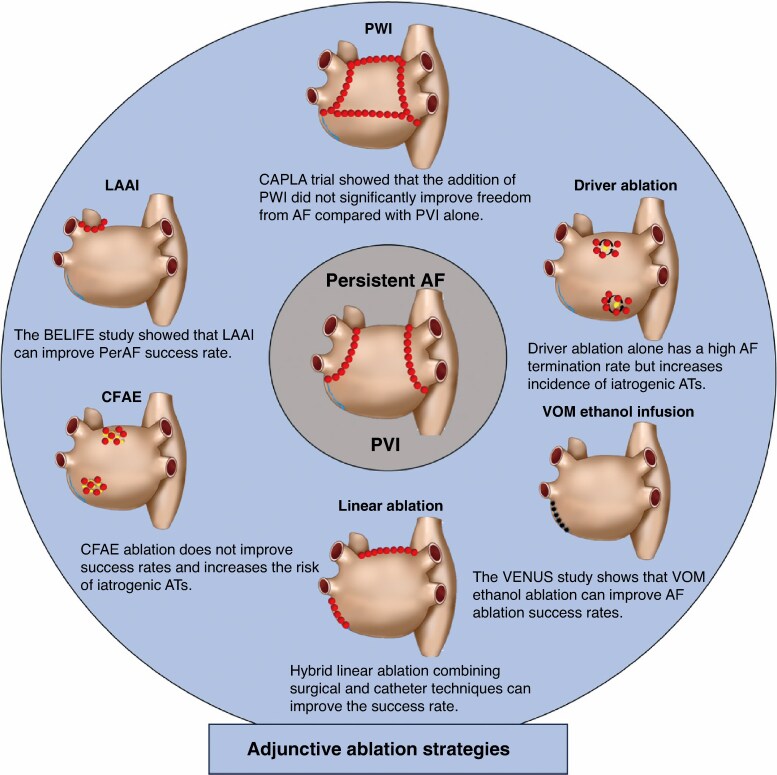
Current ablation strategies for persistent AF. AF, atrial fibrillation; AT, atrial tachycardia; LAAI, left atrial appendage isolation; PWI, posterior wall isolation; VOM, vein of Marshall.

In previous study designs, PVI alone was often required as a control group. It is generally accepted that PVI alone is insufficient for treating persistent AF.^35,56^ Persistent AF inherently involves complex mechanisms extending beyond the pulmonary veins, including structural and electrical remodelling of the atria.^90^ Although additional ablation strategies are not explicitly stated in the guidelines,^67,91^ using PVI alone as a control group is considered inconsistent with clinical practice because it does not address the broader aspects of AF’s maintenance mechanism. Therefore, a flaw in prior studies is the insufficient extent of ablation beyond the pulmonary veins, necessitating more extensive ablation targeting the mechanisms sustaining AF. Another significant flaw in previous clinical studies on persistent AF is the inconsistency in defining ablation endpoints. Theoretically, if AF terminates during ablation, it indicates that the intervention has targeted the maintenance mechanism of persistent AF. However, the vast majority of studies lack uniformity in defining ablation endpoints, with immediate termination during the procedure not widely accepted as an endpoint.^92^ This inconsistency contributes to the low positive results observed in previous clinical studies, as the lack of a standardized approach undermines the comparability and reproducibility of findings (*Figure 2*).

To date, only a few randomized controlled studies, such as BELIEF, VENUS, and FLOW-AF, have yielded positive results in the treatment of persistent AF.^56,93,94^ These studies have succeeded for several reasons: firstly, comprehensive ablation techniques to specifically target both the triggers and substrates responsible for initiating and perpetuating AF; secondly, durable anatomical linear lesions were achieved through ablation strategies, which are crucial for reducing iatrogenic atrial flutter; thirdly, by utilizing precise mapping techniques and defining clear ablation endpoints, treatments were tailored to individual patients, addressing specific mechanisms that sustain AF. These integrated approaches have significantly enhanced treatment efficacy and contributed to improved clinical outcomes for patients with AF.

These studies underscore the importance of a unified approach to defining and measuring success in ablation procedures. Future research should incorporate additional electro-anatomical guided strategies beyond PVI and establish consistent endpoints and methodologies. By doing so, clinical studies can provide more reliable and ethically sound evidence, ultimately enhancing the management and outcomes of patients with persistent AF.

## Proposed new stepwise ablation protocol for persistent atrial fibrillation

To address the current issues with the design and inconsistent ablation endpoints in catheter ablation studies for persistent AF, we conducted a multicentre randomized clinical trial. The study involved 450 patients, randomly assigned to one of the three groups: anatomical guided ablation (ANAT group), electrogram-guided ablation (EGM group), and extensive electro-anatomical guided ablation (EXT group). Results showed that 72% of patients in the EXT group were free from AF recurrence at 12 months, compared with 64% in the EGM group and 54% in the ANAT group. Safety endpoints were similar across all three groups, indicating that the more effective strategy does not compromise patient safety.^58^ An ablation strategy addressing electro-anatomical substrates is the current optimal ablation method for persistent AF. To further investigate whether the termination of AF during the procedure indicates a favourable outcome of persistent AF ablation, we conducted a propensity score matching study.^95^ This study found that patients whose AF terminated during the procedure had a significantly higher success rate at 12 months compared with those whose AF did not terminate. Therefore, AF termination is a reliable ablation endpoint.

Based on the aforementioned study results, we propose a new stepwise ablation protocol for persistent AF. The specific steps are as follows:

Initiate with the electrical isolation of the pulmonary veins, followed by linear ablation, including creation of a roof line across the left atrium and a mitral isthmus line from the endocardium in the left atrium. If a peri-mitral block is not achieved, perform an ethanol infusion in the VOM. Conduct spatial-temporal mapping of the EGM to identify specific ablation targets. Targeted regions include areas where EGMs exhibit: spatial-temporal dispersion activation across at least three adjacent bipoles with local AF cycle length (AFCL) ≤ mean AFCL, locally short cycle length activity, high-frequency potentials, and focal activity. Continue ablation until AF is terminated. If AF persists, perform repeated mapping and continue ablation as necessary. For recurrent persistent AF, the aforementioned protocol should also be followed. Presently, there is no consensus on the ablation strategy for recurrent persistent AF.

**Figure 3:**
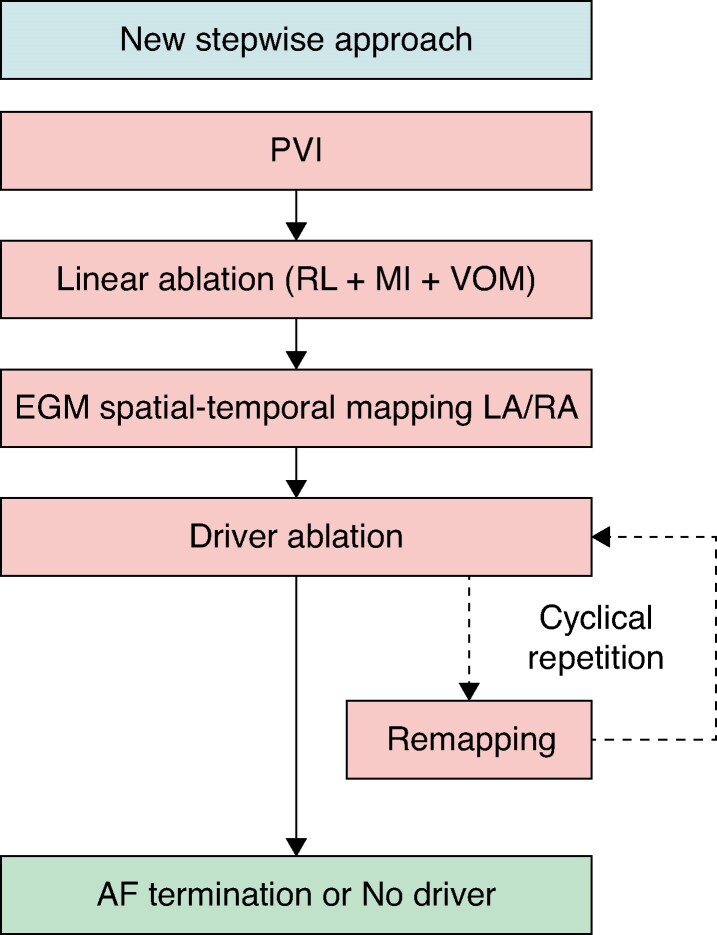
The new stepwise approach protocol. AF, atrial fibrillation; LA, left atrium; EGM, electrogram; MI, mitral isthmus; PVI, pulmonary vein isolation; RA, right atrium; RL, roof line; VOM, vein of Marshall.

This protocol emphasizes the importance of integrating electro-anatomical guidance and aiming for AF termination to optimize patient outcomes in persistent AF (*Figure 3*).

## Pulsed field ablation: adding wings to the tiger

Pulsed field ablation (PFA) is an innovative pioneering ablation technique employing non-thermal energy to disrupt cell membranes, providing targeted treatment with minimal collateral damage compared with radiofrequency ablation. Initial trials such as IMPULSE and PEFCAT in patients with paroxysmal AF demonstrated durable PVI and safety.^96^ Subsequent trials, including PULSED AF and INSPIRE, have evaluated the safety and efficacy of PFA systems, yielding promising initial results indicating acute PVI and potential combined approaches with radiofrequency ablation.^97,98^ In patients with persistent AF, PFA achieved complete PVI and posterior left atrial wall isolation with high success rates.^99^ In terms of long-term efficacy and safety, recent studies (ADVENT, ADMIRE, and MINIFEST-17K) have shown results comparable with traditional thermal ablation techniques.^100–102^ Recently, the SPHERE PerAF trial established that in patients with persistent AF, the utilization of an all-in-one high-density mapping and ablation catheter equipped with a dual-energy ablation system demonstrated superior effectiveness compared with conventional standard treatments.^103^ Furthermore, recent studies have shown that using a pentaspline PFA catheter in a tailored multi-stage ablation strategy achieved AF termination in 95.8% of persistent and long-standing persistent AF patients, with a single-procedure success rate of 74.6% and AF-free survival of 89.2% after 1 year of follow-up.^104^ These results underscore PFA's superiority as an effective and safe approach for substrate modification in complex AF cases. Both linear ablation and driver-targeted ablation techniques benefit from PFA, providing advantages in transmural lesion creation and safety. This positions PFA as a valuable tool in achieving successful ablation of persistent AF. With the rapid advances in PFA technology and new iterations targeting thicker myocardium, electrophysiologists may be cautiously optimistic about positive impact of PFA on AF ablation.

In summary, persistent AF ablation is finally glimpsing the light ahead after more than two decades of advancements. Recent innovations in ablation strategies and technologies have markedly improved success rates and patient outcomes. Our findings highlight the efficacy of extensive electro-anatomical guided ablation and the promising potential of PFA. These advancements offer a brighter future for the management of persistent AF, transforming a glimmer of hope into a sustained vision of success.

## Supplementary Material

euaf037_Supplementary_Data

## Data Availability

The data underlying this article are available in the article and in its online Supplementary material.
