# Supplementary material for: Persistent atrial fibrillation ablation: glimpsing the light ahead?
Source: Europace. 2025 Feb 17;27(6):euaf037. doi: 10.1093/europace/euaf037 (PMC12187518; doi:10.1093/europace/euaf037)
Supplement: euaf037_Supplementary_Data [file euaf037_supplementary_data.docx]

Table S1. List of randomized controlled trials

| **Study** | **Sample Size** | **Intervention** | **Inclusion** | **Study Design** | **Clinical trials registry ID** |
| --- | --- | --- | --- | --- | --- |
| Willems 2006 | 62 | PVI vs PVI+Substrate modification | Persistent AF | RCT | - |
| Elayi 2008 | 144 | PVI+CFAE vs PVI | Longstanding permanent AF | RCT | - |
| Estner 2008 | 77 | CFAE+PVI vs CFAE | Persistent AF | RCT | - |
| Oral 2008 | 119 | PVI + CFAE vs PVI | Longstanding persistent atrial fibrillation | RCT | - |
| Mikhaylov 2010 | 34 | PVI + linear vs PVI linear + LA septal line | Longstanding persistent AF | RCT | - |
| Elayi 2011 | 98 | PVI+CFAE vs PVI | Longstanding persistent AF | RCT | - |
| Estner 2011 | 116 | PVI+linear vs PVI+CFAE | Persistent AF | RCT | - |
| Hunter 2011  CFAE AF | 20 | PVI+CFAE vs PVI | Persistent AF | RCT | NCT00894400 |
| Dixit 2011  RASTA | 156 | PVI vs PVI+ substrate modification | Persistent AF | RCT | NCT00379301 |
| Pokushalov 2013 | 264 | PVI+LL vs PVI+GP ablation | Persistent/long-standing persistent AF | RCT | - |
| Wang 2014 | 124 | Substrate modification vs stepwise ablation | Longstanding persistent AF | RCT | - |
| Han 2014 | 119 | PVI+linear vs PVI+CFAE | Longstanding persistent AF | RCT | - |
| Wang 2014 | 210 | PVI+Line+CFAE vs PVI+Line+CTI vs PVI+ CFAE | Longstanding AF | RCT | - |
| Lin 2014 | 120 | Limited versus extensive atrial substrate modification (CFAE) | Non-paroxysmal AF | RCT | - |
| Kim 2015 | 120 | PVI+line+PWI vs PVI+line | Persistent AF | RCT | - |
| Dong 2015 | 146 | ‘2C3L’ strategy vs Stepwise strategy | Persistent AF | RCT | - |
| Wong 2015 | 65 | PVI+linear+CFAE vs PVI+linear ablation | Persistent AF | RCT | NCT01711047 |
| Julia Vogler 2015 CHASE AF | 205 | PVI +CFAE+Linear vs PVI | Persistent AF | RCT |  |
| Verma 2015  STAR AF II | 589 | PVI vs PVI+CFAE vs PVI +line | Persistent AF | RCT | NCT01203748 |
| Sheldon 2016 MAGIC-AF | 200 | CFAE ablation with ibutilide VS PVI | Chronic AF | RCT | NCT01014741 |
| Bassiouny 2016 | 90 | PVI vs PVI+CFAE | Persistent AF | RCT | NCT02429648 |
| Luigi Di Biase 2016 BELIEF | 173 | Standard Ablation + LAA isolation vs Standard Ablation | Longstanding persistent AF | RCT | NCT01362738 |
| Lin 2016 | 68 | Driver +PVI vs PVI +CFAE | Persistent AF | RCT |  |
| Kim 2017 | 137 | PVI+Linear vs PVI+Linear+CFAE | Longstanding Persistent AF | RCT | NCT02175043 |
| Ammar-Busch 2017 | 90 | PVI+CFAE vs PVI+ CFAE+Linear | Persistent AF | RCT | - |
| Thomas Fink 2017  Alster-Lost-AF | 124 | PVI or PVI plus substrate modification. | Persistent and longstanding Persistent AF | RCT | NCT00820625 |
| Yang 2017  STABLE-SR | 229 | Substrate-based modification vs electrogram-based modification | Non-paroxysmal AF | RCT | NCT01761188 |
| Yu 2017 | 113 | PVI vs PVI + linear ablation | Persistent Atrial Fibrillation | RCT | NCT02176616 |
| Pappone 2018 | 81 | Driver ablation+PVI vs PVI | Persistent AF | RCT | NCT02571218 |
| Mohant 2018  OASIS | 87 | FIRM-ablation only vs FIRM+PVAI ablation vs PVI+PW+NPV ablation | Non-paroxysmal AF | RCT | NCT02533843 |
| Lee 2019 | 217 | CPVI vs CPVI + PWI | Persistent AF | RCT | NCT02721121 |
| Lee 2019 | 150 | PVI+CFAE-guided focal ablation vs PVI+CFAE-guided linear ablation | Non-paroxysmal AF | RCT | - |
| Lin 2019 | 142 | Dispersion-guided ablation+PVI vs PVI+CFAE+linear | Persistent AF | RCT | - |
| Haldar 2020  CASA-AF | 120 | Catheter ablation vs. thoracoscopic surgical ablation (PVI+roof+inferior) | Longstanding persistent AF | RCT | NCT02755688. |
| Pak 2020 | 114 | PVI vs PVI+POBI | Persistent AF | RCT | NCT02176616. |
| Yamaji 2020 | 111 | PVI+EP test-guided adjunctive PWI vs PVI | Persistent AF | RCT | - |
| Yao 2020  CLEAR-AF | 214 | PVI + LA roof line+ LA anterior wall line vs PVI + LA roof line | Persistent AF | RCT | NCT02892162 |
| Valderrábano 2020  VENUS | 350 | Catheter ablation alone vs Catheter ablation + vein of Marshall ethanol infusion | Persistent AF | RCT | NCT01898221 |
| Aryana 2020 | 110 | PVI vs PVI + PWI (CBA) | Persistent AF | RCT | NCT03057548 |
| DeLurgio 2020  CONVERGE | 153 | Hybrid Convergent procedure vs endocardial catheter ablation | Persistent and longstanding persistent AF | RCT | NCT01580124 |
| Baek 2021 | 170 | PVI+dominant frequency guided ablation vs PVI | Persistent AF | RCT | KCT0003613 |
| Inoue 2021  EARNEST-PVI | 497 | PVI vs PVI + CFAE and/or linear ablation | Persistent AF | RCT | NCT03514693 |
| Hu 2021 | 125 | PVI+Driver ablation vs PVI | Persistent AF with obesity | RCT | - |
| Hwang 2021 | 50 | PVI + CFAE vs PVI | Persistent Atrial Fibrillation | RCT | - |
| Honarbakhsh 2022  TARGET-AF1 | 40 | Driver ablation vs PVI | Persistent AF | RCT | - |
| Jinhee Ahn 2022 | 100 | PVI vs PVI+PWI(CBA) | Persistent AF | RCT | - |
| Marrouche 2022  DECAAF II | 843 | MRI-guided fibrosis ablation plus PVI vs PVI | Persistent AF | RCT | NCT02529319 |
| Yang 2022  Stable SR II | 300 | CPVI Alone vs CPVI +Electrophysiological Substrate Ablation | Persistent AF | RCT | NCT03448562 |
| Ahn 2022 | 100 | PVI vs PVI+PWI (CBA) | Persistent AF | RCT | KCT0004149 |
| Yan Huo 2022  ERASE-AF | 324 | PVI vs PVI + substrate modification | Persistent AF | RCT | NCT02732626 |
| Bastian Kaiser 2023 | 100 | PVI+substrate modification (low-voltage areas) vs PVI+linear ablation | Persistent AF | RCT | - |
| Masuda 2023  EARNEST-PVI | 496 | PVI-alone vs PVI +linear+CFAE | Persistent AF | RCT | UMIN000019449 |
| Jeremy William 2023 | 98 | PVI+PWI vs PVI | Persistent AF | RCT (Hoc secondary analysis) | ACTRN12616001436460 |
| Lin 2023 | 170 | PVI+Driver vs PVI | Persistent AF | RCT | NCT05333952 |
| David Chieng 2023 | 210 | PVI+PWI vs PVI | Persistent AF with heart failure | RCT (Secondary analysis) | ACTRN12616001436460 |
| Doll 2023  CEASE-AF | 154 | Hybrid epi- endocardial ablation vs endocardial ablation | Persistent and longstanding persistent AF | RCT | NCT02695277 |
| Xu 2023 | 120 | LA+RA ablation vs LA ablation | Persistent AF with Right Atrial Enlargement | RCT | ChiCTR220056844 |
| Heijden 2023  HARTCAP-AF | 41 | Hybrid Ablation vs Catheter Ablation | Persistent AF | RCT | NCT02441738 |
| Kistler 2023  CAPLA study | 338 | PVI vs PVI+PWI | Persistent AF | RCT | ACTRN12616001436460 |
| Bulava 2024  SURHYB | 231 | Hybrid Group vs Surgery Group | Non-paroxysmal AF | RCT | cz-020420181253 |
| Dhanunjaya 2024 | 610 | LAA ligation+PVI vs PVI alone. | Non-paroxysmal AF | RCT | NCT02513797 |
| Kaige Li 2024 | 450 | anatomical guided ablation vs electrogram guided ablation vs extensive electro-anatomical guided ablation | Persistent AF | RCT | ChiCTR2200060075 |
| Reddy 2024  FLOW-AF | 85 | PVI+source ablation vs PVI | Persistent AF | RCT | NCT04473963 |
| Takahashi 2024  MAP-AF | 98 | PVI+Cartofounder vs PVI | Persistent AF | RCT | UMIN000037569 |
| Zheng 2024 | 100 | Hybrid ablation vs stand-alone thoracoscopic surgical ablation | Non-paroxysmal AF | RCT | NCT03127423 |

PVI: Pulmonary Vein Isolation; CFAE: Complex Fractionated Atrial Electrograms; PWI: Posterior Wall Isolation; CBA: Cryoballoon Ablation; GP: Ganglionated Plexi; CTI: Cavotricuspid Isthmus; POBI: Posterior Wall Box Isolation; LAA: Left Atrial Appendage; LA: Left Atrium; RA: Right Atrium
